# Oral tranexamic acid and thrombosis risk in women

**DOI:** 10.1016/j.eclinm.2021.100882

**Published:** 2021-05-06

**Authors:** Amani Meaidi, Lina Mørch, Christian Torp-Pedersen, Oejvind Lidegaard

**Affiliations:** a Department of Gynaecology, Rigshospitalet, Faculty of Health and Medical Sciences, University of Copenhagen, Copenhagen, Denmark; b Department of Clinical Medicine, Faculty of Health and Medical Sciences, University of Copenhagen, Copenhagen, Denmark; c The Danish Cancer Society Research Center, Cancer Surveillance and Pharmacoepidemiology, Copenhagen, Denmark; d Department of Cardiology, Aalborg University Hospital, Aalborg, Denmark; e Department of Cardiology, Nordsjaellands Hospital, Hilleroed, Denmark; f Department of Public Health, University of Copenhagen, Denmark

**Keywords:** Acute myocardial infarction, Deep-vein thrombosis, Heavy menstrual bleeding, Pulmonary embolism, Thrombotic stroke, Tranexamic acid

## Abstract

**Background:**

Oral tranexamic acid is effective for heavy menstrual bleeding, but the thrombosis risk with this treatment is largely not studied.

**Methods:**

Using nationwide registries, we assessed associations between use of oral tranexamic acid and risk of deep-vein thrombosis or pulmonary embolism and arterial thrombosis in heart or brain in a nationwide historical prospective cohort of Danish women aged 15 to 49 years in the period 1996–2017. Exclusion criteria included potential confounding factors such as history of thromboembolism, anticoagulation therapy, thrombophilia, and cancer.

**Findings:**

Among 2·0 million women followed for 13·8 million person-years, 3,392 venous thromboembolisms and 4,198 arterial thromboses occurred. A total of 63,896 women (3·2%) filled 146,729 prescriptions of oral tranexamic acid during follow-up with median filled prescription per user being one of 15 g. The age-standardised incidence rate of venous thromboembolism was 11·8 (95% CI 4·6 to 30·2) per 10,000 person-years in oral tranexamic acid use compared to 2·5 (2·4 to 2·6) per 10,000 person-years in non-use. For arterial thrombosis, the age-standardised incidence rate per 10,000 person-years was 3·4 (1·1 to 10·7) among exposed compared to 3·0 (2·9 to 3·1) in non-exposed. Comparing oral tranexamic acid use with non-use, the adjusted incidence rate ratio was 4·0 (1·8 to 8·8) for venous thromboembolism and 1·3 (0·4 to 4·2) for arterial thrombosis.

Number needed to harm per five days of treatment was 78,549 women for venous thromboembolism.

**Interpretation:**

We found use of oral tranexamic acid to be positively associated with venous thromboembolism. However, number needed to harm per five days of treatment was high.

**Research in context**

**Evidence before this study**

Randomised controlled trials studying the effect of oral tranexamic acid on heavy menstrual bleeding have not been sufficiently powered to assess the risk of thrombosis with this treatment.

Two observational studies have assessed the association between oral tranexamic acid for heavy menstrual bleeding and risk of venous thromboembolism and reported contrary findings.

**Added value of this study**

This is the first nationwide cohort study on the influence of oral tranexamic acid for heavy menstrual bleeding on the risk of thrombosis, accounting for a wealth of possible confounding factors.

We report a four-fold increased incidence rate of venous thromboembolic events associated with use of oral tranexamic acid compared to non-use. However, number needed to harm per five days of treatment was 78,549 women. We found no association with arterial thrombosis.

**Implications of all the available evidence**

Although confirming the concern of an association with venous thromboembolic disease, we found venous thromboembolism to be a very rare adverse event of oral tranexamic acid when used for a short term and in generally healthy women. This supports a majority of studies reporting a favourable safety profile of the treatment of tranexamic acid for other indications.

Alt-text: Unlabelled box

## 1 Introduction

Heavy menstrual bleeding is a high-prevalent condition affecting the quality of life of women worldwide. Studies objectively measuring menstrual blood loss have reported heavy menstrual bleeding in nine to 14% of pre- and perimenopausal women, while studies based on self-assessment methods report higher frequencies, ranging between 20 and 52% [1], [2], [3], [4]. The condition negatively impacts the physical, mental, and social wellbeing of affected women and has been associated with unemployment and absence from work, emphasizing the importance of effective and safe treatment options [5], [6], [7], [8], [9].

Orally administrated tranexamic acid is a potent haemostatic agent recommended both as acute and as long-term treatment of heavy menstrual bleeding [10,11]. While the ability of oral tranexamic acid to reduce menstrual blood loss is well-established, the safety of the drug remains a concern [12]. Being an antifibrinolytic agent, tranexamic acid inhibits the lysis of fibrin. Fibrin constitutes the main component of both venous and arterial thromboses, which has led to concerns about tranexamic acid being able to stabilize an initiating thrombosis formation and thereby increase the risk of thromboembolic events [13,14]. Clinical trials on the treatment effects of oral tranexamic acid on heavy menstrual bleeding, most of them including less of 100 participants, have not been sufficiently powered to study the association with thromboembolism, and observational studies on the topic are sparse, small, and inconclusive [12,[15], [16], [17], [18], [19]].

The influence of tranexamic acid on thromboembolic risk has primarily been investigated in patients suffering from other bleeding conditions than heavy menstrual bleeding with conflicting results in trauma patients, no apparent risk when given for prevention of blood loss following vaginal delivery, and an increased risk in patients with acute gastrointestinal bleeding [20], [21], [22], [23].

Aiming to clarify the safety of tranexamic acid as a treatment option for heavy menstrual bleeding, this pharmacoepidemiologic study follows a nationwide cohort of women aged 15 to 49 years with the objective of assessing the association between use of oral tranexamic acid and the risk of venous thromboembolism and arterial thrombosis.

## 2 Methods

### 2.1 Study design and population

We conducted a nationwide historical prospective cohort study using information from the Civil Registration System, the National Registry of Causes of Death, the National Patient Registry, the Danish National Registry of Medicinal Product Statistics, the Danish Medical Birth Registry, the Registry of Legally Induced Abortions, and the Population Education Registry [24], [25], [26], [27], [28], [29], [30]. A personal identification number given to all Danish citizens at birth or immigration was used to reliably link data from these registries.

We followed women aged 15 to 49 years from January 1st, 1996, through December 31st, 2017. Women were only included in the cohort if they at entry did not have a history of any kind of venous or arterial thrombosis, hypertension, diabetes, cancer, liver disease, thrombophilia, hemophilia, thyroid disease, endometriosis, polycystic ovary syndrome, hereditary angioedema, hysterectomy, bilateral oophorectomy, blood transfusion, use of anticoagulative medication, use of hormone therapy, use of ulipristal acetate for treatment of uterine fibroids, and use of haemostatics other than tranexamic acid (Supplementary Appendix Table S1). Immigrated women had to have lived in Denmark for at least one year before entry.

Women were followed until the end of the study period, the age of 50 years, date of emigration from the country, time of death, or time of the experience of one of the criterions of exclusion, whichever came first.

This national cohort study was approved by the Danish Data Protection Agency and the Danish Health Data Board. Registry-based studies are not subject to ethics approval in Denmark.

AM and CTP had access to the raw data from January 2017.

### 2.2 Oral tranexamic acid

If a woman filled a prescription of oral tranexamic acid, she was considered a user. The Danish National Registry of Medicinal Product Statistics holds information on all filled prescriptions in Denmark since 1995 [26]. Searching the Anatomical Therapeutic Chemical code of tranexamic acid (B02AA02) and conditioning on oral administration, we identified users of oral tranexamic acid. In Denmark, oral tranexamic acid cannot be purchased over-the-counter.

Oral tranexamic acid is primarily used in the acute management of heavy menstrual bleeding. One gram three times a day in five days is the most frequently prescribed dosage [31]. The majority of women only fill a single prescription of 15 gs, corresponding to the standard treatment of one menstrual bleeding [31]. Thus, most users of oral tranexamic acid in current study are treated for five days [31].

To account for a potential delay in treatment initiation, six exposure windows were assessed from the date of filled prescription; 1) five days initiated at date of filled prescription (exposure time of five days), 2) five days initiated sometime in the first week from date of filled prescription (exposure window of five days plus one week), 3) five days initiated sometime in the two weeks following date of filled prescription (exposure window of five days plus two weeks), 4) five days initiated sometime in the three weeks from date of filled prescription (exposure window of five days plus three weeks), 5) five days initiated sometime in the four weeks following date of filled prescription (exposure window of five days plus four weeks), 6) five days initiated sometime in the eight weeks following date of filled prescription (exposure window of five days plus eight weeks), respectively. The exposure window that provided the highest incidence rate of thrombosis was considered the most reliable definition.

### 2.3 Thromboembolic events

A woman was considered to experience an event of interest, if she received a diagnosis (classified according to *The International Classification of Diseases, 10th revision*) of deep-vein thrombosis (I801–3), pulmonary embolism (I26), acute myocardial infarction (I21), or thrombotic stroke (I63–4, except I636) in the National Patient Registry or in the National Registry of Causes of Death [25,27]. Diagnoses of deep-vein thrombosis and pulmonary embolism given only in the emergency department have low validity and were therefore not considered. A sensitivity analysis included only diagnoses of venous thromboembolisms followed by a filled prescription of relevant anticoagulation therapy within six months from date of diagnosis. Most women receive initial anticoagulation therapy at the hospital without prescription, which is why a six-month window is needed to capture prescription redemption.

### 2.4 Confounding factors

A woman was temporarily censored during pregnancy and for six months after delivery and twelve weeks after other types of pregnancy terminations (Supplementary Appendix Table S1). The Danish Medical Birth Registry and The Registry of Legally Induced Abortions hold information on all births and induced abortions undergone in Denmark since 1973, respectively, including date of birth/abortion and gestational age at birth/abortion, allowing the calculation of estimated date of conception and thereby the temporarily censoring of women during and following pregnancy [28,29]. Similarly, miscarriages, extrauterine pregnancies, and pregnancies with unknown locations diagnosed or treated in a hospital setting are registered in the National Patient Registry with date of termination and gestational age at that time point [27]. Miscarriages diagnosed and treated in the primary health care sector were not detectable. The completeness of pregnancy registrations in the Medical Birth Registry, The Registry of Legally Induced Abortions, and in the National Patient Registry is expected to be very high, considering that the recordings are mandatory and necessary for the departments to receive governmental payment for their services.

If a woman had uterine surgery (other than a hysterectomy) or any kind of surgery demanding hospitalization for more than one day, she was temporarily censored in eight weeks from day of discharge (Supplementary Appendix Table S1). Furthermore, a woman was also temporarily censored during use of hormonal contraception, other systemic progestogens, or nonsteroidal antiinflammatory drugs (Supplementary Appendix Table S1).

A woman was considered a user of hormonal contraception, if she filled a prescription of such. In Denmark, purchase of any hormonal contraceptive demands a prescription and is registered in the National Registry of Medicinal Product Statistics [26]. Time exposed to hormonal contraception was defined from date of filled prescription. For hormonal contraceptives administrated orally, by patch, injection, or vaginal ring, duration of use was determined by amount of defined daily doses purchased. According to previous studies, duration of usage of intrauterine systems and implants was assumed to be one year less of the maximal possible usage to account for possible early discontinuation [32,33]. All exposure periods were extended by 28 days to account for delays in initiation of use.

Due to the different possible dosages of systemic cyclic progestogen-only therapy, we used the program medicinMacro, available in the R package “github/tagteam/heaven”, to calculate the most likely time and duration of treatment. The calculation was based on information on time of purchase and amount purchased, provided by the Registry of Medicinal Product Statistics, as well as information on minimal, maximal, and default dosage, defined according to the recommendations provided in the summary of product characteristics [26]. The program assumed the default dosage at the initiation of treatment. If more than one purchase occurred, the program calculated whether treatment could have been continuous with minimal dosage or maximal dosage based on data of up to five previous purchases. All calculated treatment periods were extended with 28 days to account for possible delays in treatment initiation.

Time exposed to nonsteroidal antiinflammatory drugs was assumed to be one week from a filled prescription of any such drug.

Temporarily censored time periods did not contribute with person-time or thromboembolic events in the analyses, and women experiencing an event, death, emigration, or any exclusion criterion during a temporarily censored time period were permanently censored from that time point.

Time-updated information on age, calendar time, and educational level was also retrieved (Supplementary Appendix Table S1). Information on body-mass index and smoking status was available for parous women only as it was recorded at first visit to a midwife during pregnancy (Supplementary Appendix Table S1).

### 2.5 Statistical analysis

Poisson regressions were used to estimate adjusted incidence rate ratios with 95% confidence intervals of venous thromboembolism and arterial thrombosis according to use of oral tranexamic acid. The reference group was non-use in all analyses.

All reported incidence rate ratios were adjusted for age and calendar time in five-year intervals as well as educational level (elementary school only, secondary school only, skilled worker, theoretical education, theoretical education with research qualifications).

Age-standardised incidence rates of venous thromboembolism and arterial thrombosis were calculated according to use of oral tranexamic acid using the age distribution of the entire cohort as the standard. Age-standardised absolute rate differences (age-standardised incidence rates_exposed_ - age-standardised incidence rates_unexposed_) were then calculated. All rates were reported per 10,000 person-years. Number needed to harm per five days of treatment was also assessed (1/[*age*-*standardised incidence rates_exposed_* - *age*-*standardised incidence rates_unexposed_*].

As a supplement, number of oral tranexamic acid users and number of thromboses during use were explored among excluded and censored women as well as among women temporarily censored.

Data were managed with SAS software, version 9.4 (SAS Institute), while analysed using R software [34].

### 2.6 Role of the funding source

The research was supported by the Danish Heart Foundation, which had no role in any part of the scientific process.

## 3 Results

### 3.1 Characteristics of the study population

**Fig. 1:**
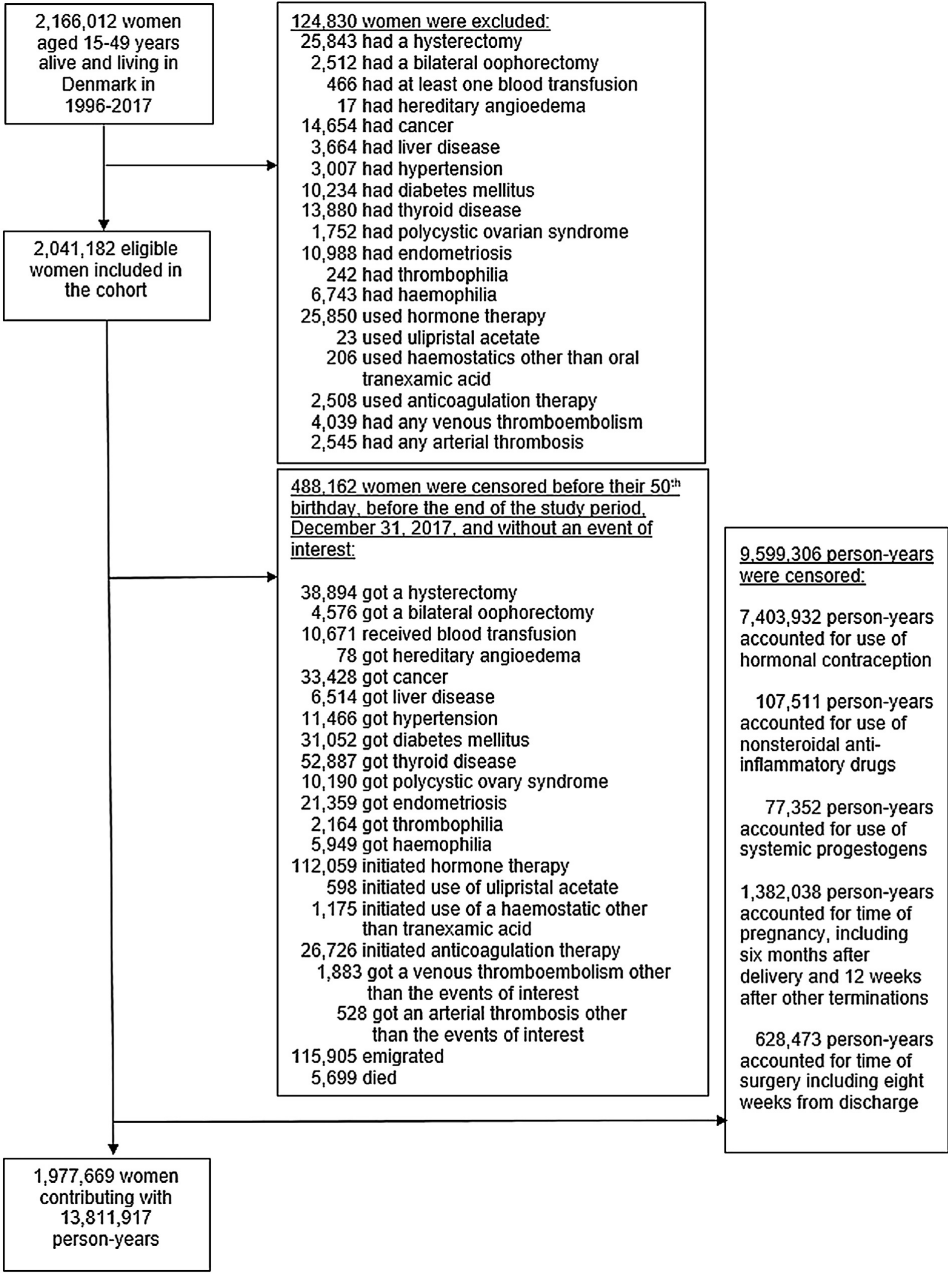
*Flow-diagram of the formation of the study cohort*.

A total of 1977,669 women were included in the cohort (Fig. 1) and followed up for 13,811,917 person-years with a median (first to third quantile) follow-up time of 5·3 years (2·1 to 10·8 years). During this period, 3392 women had a first venous thromboembolism of which 932 (27·5%) were coded as pulmonary embolisms, and 4198 had a first arterial thrombosis of which 1343 (32·0%) were coded as acute myocardial infarctions. Of all observed diagnoses of venous thromboembolisms, 1433 diagnoses (42·2%) were confirmed by a filled prescription of relevant anticoagulation therapy within six months from diagnosis.

A total of 63,896 (3·2%) women filled at least one prescription of oral tranexamic acid during follow-up. Characteristics of the cohort according to use of oral tranexamic acid are shown in Table 1.

**Table 1:** Characteristics of the study population according to use of oral tranexamic acid.

	Non-use of oral tranexamic acid	Use of oral tranexamic acid*
	*No. of Person-Yr (Column Percentage)*	
Total	13,809,120 (100·0)	2797 (100^·^0)
Age group (years)		
15–19	2035,257 (14^·^7)	54 (1^·^9)
20–24	1342,642 (9^·^7)	49 (1^·^8)
25–29	1528,323 (11^·^1)	101 (3^·^6)
30–34	1886,799 (13^·^7)	216 (7^·^7)
35–39	2247,529 (16^·^3)	462 (16^·^5)
40–44	2432,309 (17^·^6)	803 (28^·^7)
45–49	2336,261 (16^·^9)	1112 (39^·^8)
Calendar time		
1996–1999	2178,716 (15^·^8)	808 (28^·^9)
2000–2004	3288,351 (23^·^8)	763 (27^·^3)
2005–2009	3063,439 (22^·^2)	612 (21^·^9)
2010–2014	2962,300 (21^·^5)	431 (15^·^4)
2015–2017	2316,314 (16^·^8)	183 (6^·^5)
Educational level		
Elementary school only	6679,445 (48^·^4)	878 (31^·^4)
Secondary school only	1086,276 (7^·^9)	147 (5^·^3)
Skilled worker	3503,158 (25^·^4)	1122 (40^·^1)
Theoretical education	1923,300 (13^·^9)	521 (18^·^6)
Theoretical education with research qualifications	616,941 (4^·^5)	129 (4^·^6)
Body-mass index (kg/m^2^)		
<18^·^5	57,051 (0^·^4)	11 (0^·^4)
18^·^5–25	810,750 (5^·^9)	95 (3^·^4)
>25 to 30	283,220 (2^·^1)	51 (1^·^8)
>30	162,209 (1^·^2)	38 (1^·^4)
Unknown	12,495,890 (90^·^5)	2602 (93^·^0)
Smoking status		
Yes	546,774 (4^·^0)	117 (4^·^2)
No	2190,589 (15^·^9)	399 (14^·^3)
Unknown	11,071,757 (80^·^2)	2281 (81^·^6)

*Five days of exposure to oral tranexamic acid was assumed per 15 g redeemed^·^

Exposure was assumed to occur from date of filled prescription.

### 3.2 Pattern of oral tranexamic acid utilization in the study population

The 63,896 unique users of oral tranexamic acid filled a total of 146,729 prescriptions of 3064,859 gs of oral tranexamic acid during follow-up. Of these prescriptions, around 75% accounted for 15 g, 16% for 30 g, 1% for 45 g, 7% for 50 g, and 1% for 60 g. Pattern of oral tranexamic acid use according to age at prescription redemption is illustrated in Table 2. Median (first to third quantile) number of filled prescriptions per user was 1 (1 to 2), and median dose redeemed per user was 15 g (15 to 30 g), accounting for the standard treatment of one menstrual bleeding. The medians were similar across age. Of all users, 33·4% filled at least two prescriptions, and 42·7% filled at least 30 g, accounting for the standard treatment of at least two menstrual bleedings. Use of oral tranexamic acid peaked in women aged 45 to 49 years.

**Table 2:** Oral tranexamic acid utilization according to age.

Age	Users		First-time users		Prescriptions			Prescribed dose		
group (years)	*n*	%	*n*	%	*N*	%	*n* per user*	Gram	%	gram per user*
15–19	1578	2^·^2	–	–	2800	1^·^9	1 (1–2)	59,605	1^·^9	15 (15–30)
20–24	1923	2^·^6	1825	94^·^9	2683	1^·^8	1 (1–1)	53,421	1^·^7	15 (15–15)
25–29	3828	5^·^3	3648	95^·^3	5637	3^·^8	1 (1–1)	110,168	3^·^6	15 (15–30)
30–34	7216	9^·^9	6746	93^·^5	12,283	8^·^4	1 (1–2)	236,541	7^·^7	15 (15–30)
35–39	12,384	17^·^0	11,100	89^·^6	24,534	16^·^7	1 (1–2)	506,112	16^·^5	15 (15–30)
40–44	19,675	27^·^1	17,119	87^·^0	41,771	28^·^5	1 (1–2)	880,315	28^·^7	15 (15–45)
45–49	26,059	35^·^9	21,880	84^·^0	57,021	38^·^9	1 (1–2)	1218,697	39^·^8	15 (15–45)
**Total**	72,663	100^·^0	63,896	87^·^9	146,729	100^·^0	1 (1–2)	3064,859	100^·^0	15 (15–30)

*Median (first to third quantile).

### 3.3 The adjusted incidence rate ratios of thrombosis associated with use of oral tranexamic acid

During follow-up, between the date of filling a prescription of oral tranexamic acid and up to eight weeks from that date, 26 thromboembolic events were observed among the users of tranexamic acid, 16 venous thromboembolisms (eight confirmed) and ten arterial thromboses (Table 3).

**Table 3:** Number of observed thromboembolic events among women purchasing oral tranexamic acid within eight weeks from a filled prescription according to age at time of purchase.

Age group (years)	Users of oral tranexamic acid	Number of prescriptions	Percent of prescriptions followed eight weeks or until event*	Events within eight weeks#	
				Venous"	Arterial
15–34	14,545	23,403	96·2	3 (0)	0
35–49	58,118	123,326	92·7	13 (8)	10
Total	72,663	146,729	93·2	16 (8)	10

# From day of filled prescription of oral tranexamic acid. *For the remaining percentages, women were censored before the end of the eight-week period for an exclusion criterion other than the occurrence of a thromboembolic event of interest. "Reported in the () is the number of diagnoses confirmed by a filled prescription of relevant anticoagulation therapy within six months from diagnosis.

The incidence rate of both venous and arterial thrombosis peaked, when time window for exposure to oral tranexamic acid was defined as five days plus one week from date of filled prescription.

As compared with non-use, use of oral tranexamic acid was associated with a significant increased incidence rate ratio of venous thromboembolism, 4·0 (95% CI 1·8 to 8·8), and an incidence rate ratio of arterial thrombosis of 1·3 (0·4 to 4·2, Table 4).

**Table 4:** Adjusted incidence rate ratios of venous and arterial thrombosis according to use of oral tranexamic acid in women aged 15–49 years.

		Venous thromboembolism			Arterial thrombosis		
Use of oral tranexamic acid	No. of Person-Yr	No.#	Age-standardised Incidence rate (95% CI) *events/10,000 person-yr*	Adjusted Incidence Rate Ratio* (95% CI)	No.	Age-standardised Incidence rate (95% CI) *events/10,000 person-yr*	Adjusted Incidence Rate Ratio* (95% Ci)
No	13,807,520	3386 (1430)	2^·^5 (2^·^4 to 2^·^6)	Reference	4195	3^·^0 (2^·^9 to 3^·^1)	Reference
Yes	4397	6 (3)	11^·^8 (4^·^6 to 30^·^2)	4^·^0 (1^·^8 to 8^·^8)	3	3^·^4 (1^·^1 to 10^·^7)	1^·^3 (0^·^4 to 4^·^2)

# Reported in () is the number of diagnoses confirmed by a filled prescription of relevant anticoagulation therapy within six months from diagnosis. *Adjusted for age, calendar time, and educational level.

A sensitivity analysis of only confirmed venous thromboembolisms did not materially change the main estimate of the association between use of oral tranexamic acid and venous thromboembolism, 4·3 (1·4 to 13·3).

In total, 329,375 parous women had information on both body-mass index and smoking status. Of these women, 6080 filled at least one prescription of oral tranexamic acid. No venous thromboembolisms or arterial thromboses occurred in the time window of five days plus one week following date of filled prescription among these users. Thus, we were not able to estimate incidence rate ratios adjusted for body-mass index and smoking status.

### 3.4 The age-standardised incidence rates of thrombosis associated with use of oral tranexamic acid and numbers needed to harm

While the age-standardised incidence rate of venous thromboembolism was 2·5 (2·4 to 2·6) per 10,000 person-years in non-use of oral tranexamic acid, the difference in age-standardised incidence rate of venous thromboembolism between use and non-use was 9·3 (8·4 to 10·3) per 10,000 person-years; approximately one extra venous thromboembolism was diagnosed for every 78,549 women using oral tranexamic acid for five days.

For arterial thrombosis, the age-standardised incidence rate in non-use was 3·0 (2·9 to 3·1) per 10,000 person-years, and the difference between use and non-use of oral tranexamic acid was non-significant, 0·4 (−0·7 to 1·5) per 10,000 person-years, the best estimated number needed to harm being 1826,262 for five days of treatment.

All exposed women experiencing a venous thromboembolism within five days plus one week from date of filled prescription only purchased 15 g of oral tranexamic acid prior to the event.

### 3.5 Thromboses in excluded and censored users of oral tranexamic acid

Only five additional thromboses occurred in excluded and permanently censored users of oral tranexamic acid (Supplementary Appendix Table S2). Similarly, only five venous thromboembolisms were observed during concomitant use of oral tranexamic acid and combined hormonal contraception, and even fewer thromboses were observed in concomitant use with systemic progestogens or nonsteroidal antiinflammatory drugs (Supplementary Appendix Table S3).

## 4 Discussion

In this historical prospective cohort study, use of oral tranexamic acid implied an increased incidence rate of venous thromboembolism compared to non-use. However, number needed to harm per five days of treatment was high. Use of oral tranexamic acid was not found to be significantly associated with arterial thrombosis.

Oral tranexamic acid is one of the most effective medical therapies for heavy menstrual bleeding [12]. Even after the appearance of the levonorgestrel-releasing intrauterine device, oral tranexamic acid still plays an essential role in the acute management of heavy menstrual bleeding which no other currently available medical therapy can fill. It also provides a possibility of treatment for women with a current desire of pregnancy or women with contraindications against hormonal therapy and surgical treatment not possible due to a future desire of pregnancy.

In Denmark, oral tranexamic acid is primarily prescribed in the acute management of heavy menstrual bleeding, while the levonorgestrel-releasing intrauterine system is used in the long-term management [31]. As a long-term treatment option, the levonorgestrel-releasing intrauterine system has shown to be superior and is significantly more affordable compared to oral tranexamic acid [12,31]. In view of the lack of alternative medical therapies in the acute handling of heavy menstrual bleeding, our findings, based on this pattern of short-term use, is of high clinical relevance.

In women not exposed to oral tranexamic acid, we found the age-standardised incidence rate of venous and arterial thrombosis to be 3 per 10,000 person-years, respectively. The incidence rates are comparable with previous studies on the occurrence of thrombotic disease in reproductive-aged women outside the time of surgery, pregnancy, and use of hormonal contraception [32,33].

We found the age-standardised incidence rate difference of venous thromboembolism between users and non-users of oral tranexamic acid to be of the same order as the age-standardised incidence rate difference between use and non-use of combined oral contraceptives, which are used regularly to treat heavy menstrual bleeding [10,34,35]. The age-standardised incidence rate difference was smaller than that of combined oral contraceptives containing desogestrel, gestodene, drospirenone, or cyproterone, but larger than that of combined oral contraceptives including norethisterone, levonorgestrel, or norgestimate [33,35].

Recently, it was concluded that prior randomised controlled trials have not been adequately powered to study the association between oral tranexamic acid for heavy menstrual bleeding and risk of thrombotic disease [12].

Only two observational studies have assessed the association between use of oral tranexamic acid for heavy menstrual bleeding and risk of venous thromboembolism [18,19]. One indicated an increased risk, the other a protective effect [18,19]. The case-control suggesting a protective effect (odds ratio 0.55 (0.31 to 0.97)) did not adjust for any potentially confounding factors other than age and geography. In the same study population, use of oral contraceptives was associated with two-fold odds of venous thromboembolism. Oral contraceptives are also known to reduce the risk of heavy menstrual bleeding and thereby the need for oral tranexamic acid [10,12]. Thus, the apparent protective effect of tranexamic acid usage is likely explained by the lack of controlling for use of oral contraceptives [18].

This nationwide cohort study following around 2.0 million women for a total of almost 14 million person-years adds novel knowledge about the safety profile of a drug used to treat a high-frequent condition among reproductive-aged women. Through the reliable national registries, we were able to include all eligible women with no loss-to-follow-up and to eliminate a wealth of possible confounding factors such as use of hormonal contraception and pregnancy. In the Danish National Patient Registry, the positive predictive value of a first-time diagnosis of either deep-vein thrombosis or pulmonary embolism in a non-emergency department is quite high, i.e. 89% [36]. Since most patients receive free anticoagulation therapy from the hospital without prescription following a venous thromboembolism, we were only able to confirm 42% of diagnoses. Nevertheless, we found a similar incidence rate ratio after restricting to diagnoses of venous thromboembolism followed by purchase of anticoagulation therapy, confirming the validity of our main findings and a non-differential misclassification of the potential 11% false-positive diagnoses.

We were able to exclude women with known increased risk of thrombotic disease. These restrictions were made to investigate, if oral tranexamic acid, in itself, and not in the presence of thrombotic risk factors, is associated with thrombotic disease. Since the majority of reproductive-aged women are in good health without thrombogenic diseases, the restrictions do not undermine the external validity of our findings, which is further confirmed by the consistency between the observed incidence rate of thrombotic disease in the reference population in the current study and the existing literature on reproductive-aged women [32,33].

Since the effect of oral tranexamic acid on heavy menstrual bleeding is well-established in randomised trials, it would be unethical to initiate a randomised trial for the purpose of investigating a possible fatal adverse event in patients suffering from a non-fatal condition that may be managed successfully with other treatment options. Considering that we had to follow more than 60,000 oral tranexamic acid users redeeming around 150,000 prescriptions to observe 16 venous thromboembolisms and ten arterial thromboses, a randomised trial would have to be of a significant size in order to study the risk of thrombosis associated with oral tranexamic acid in women with heavy menstrual bleeding. Thus, logistically, it would be very difficult and expensive to execute such randomised controlled trial. Therefore, we believe a non-interventional post-authorization safety study, such as the one conducted, to be the best possible way to study the research question of interest.

Though the exact time of menstrual bleeding was not available, we were able to estimate the time of oral tranexamic acid use based on information on the exact date of prescription redemption and the knowledge that oral tranexamic acid primarily is being used for the acute management of heavy menstrual bleeding in Denmark [31]. However, oral tranexamic acid provided in a hospital setting was not available. This could have led to an underestimation of the association between use of oral tranexamic acid and risk of thrombosis, since women given the drug at the hospital would be assumed non-users. The potential underestimation is expected to be minor, considering that medical treatment of heavy menstrual bleeding primarily is initiated in the primary health care sector and therefore identifiable in the Registry of Medicinal Product Statistics [26].

Although being able to create a somewhat homogenous and healthy cohort of women, we were not able to study the association in heavily menstruating women exclusively. This may have led to some residual confounding by indication. Anemia may occur due to heavy menstrual bleeding and has been linked to an increased risk of venous thromboembolism [19]. However, by censoring at time of blood transfusion, we were able to exclude women with severe anemia.

We were not able to adjust for body-mass index and smoking status. The summary of product characteristics of oral tranexamic acid states a precaution of use in women with thrombogenic risk factors such as increased body-mass index and smoking. Thus, if anything, the lack of adjustment would tend to underestimate the association, since more non-users would be obese or smokers.

Finally, adjustment for body-mass index, smoking, and anemia (defined as a serum hemoglobin value of < 11.5 g/dl) was done in the previously mentioned case-control study, which only included menorrhagia patients, and which reported an, although non-significant, increased risk of venous thromboembolism associated with oral tranexamic acid usage, indicating an association independent of the factors missing in current study [19].

Despite the above-mentioned limitations, the study clearly demonstrates that in a population of healthy women, venous thromboembolism and arterial thrombosis are maximally very rare adverse events of temporarily, short-lasting use of oral tranexamic acid, which is comparable with the risk attached to use of combined oral contraception.

Further study is warranted to determine the association between use of oral tranexamic acid and risk of thrombosis in women with known risk factors for venous thromboembolism and arterial thrombosis and in prolonged use of oral tranexamic acid.

## CRediT authorship contribution statement

**Amani Meaidi:** Formal analysis, Writing – review & editing. **Lina Mørch:** Formal analysis, Writing – review & editing. **Christian Torp-Pedersen:** Formal analysis, Writing – review & editing. **Oejvind Lidegaard:** Formal analysis, Writing – review & editing.

## Declaration of Competing Interest

AM reports grants from The Danish Heart Foundation during the conduct of the study.

LSM received a grant from Novo Nordisk for studies not related to the current study.

CTP received grants from Bayer and Novo Nordisk for studies not related to the current study.

OL declares no competing interests.

## Appendix Supplementary materials

Image, application 1
