# Supplementary material for: Oral tranexamic acid and thrombosis risk in women
Source: eClinicalMedicine. 2021 May 6;35:100882. doi: 10.1016/j.eclinm.2021.100882 (PMC8176123; doi:10.1016/j.eclinm.2021.100882)
Supplement: Supplementary file 1 [file mmc1.docx]

**Supplementary Appendix**

**Table S1:** *Identification and definition of as well as rationale for variables used in the study design*

**POA: period of availability; ICD-10 & -8: The International Classification of Diseases and Related Health Problems, 10th & 8th revision; NOMESCO: The Nordic Medico-Statistical Committee Classification of Surgical Procedures; ATC: The Anatomical Therapeutic Chemical Classification System*

| **Variables** | **Data source** | **POA** | **Classification** | **Definition** | **Reason** |
| --- | --- | --- | --- | --- | --- |
| ***Exclusion/Censoring criteria*** | | | | | |
| Any venous thromboembolism | The National Registry of Patients | 1977-2017 | ICD-10 & -8 | G951F, H348E, H348F, I26, I636, I676, I8001-9, I81-2, O032, O037, O042, O047, O072, O077, O082, O223, O225, O882, O871, O873, 28944, 321, 37703, 450, 45100, 45108-9, 45190, 45192, 45199, 452-3, 59323, 671, 67399 | Possible confounding factor: Associated with an increased risk of outcome and potentially with a decrease use of exposure due to the precaution of usage in women with thrombogenic risk factors |
| Any arterial thrombosis |  |  |  | G951G, H341A, H341B, I21, I240, I513, I630-5, I638, I639, I64, I74, K550C, K550H, N280A, N280, 37702, 410, 433-4, 444 |  |
| Hysterectomy |  |  | The Danish Classification of Surgical Procedures &  NOMESCO | KLCC10, KLCC11, KLCC20, KLCD, 61000, 61020, 61040, 61050, 61100, 72110, 72230, 72650 | Not a part of the target population of the research question of interest |
| Bilateral oophorectomy |  |  |  | KLAE2, KLAF1, 60120, 60121, 60320, 70210, 70310 |  |
| Second unilateral oophorectomy |  |  |  | KLAE1, KLAF0,  60100, 60300 ,60101 ,70200 |  |
| Hormone Therapy | The Prescrip-tion Registry | 1995-2017 | ATC code | G03C, G03F, G03HB01 (only the drug by the product name Climen) | Possible confounding factor: Associated with an increased risk of outcome and potentially with exposure use |
| Ulipristal acetate |  |  |  | G03XB02 | Possible confounding factor: Treats uterine fibroids and thereby associated with exposure and potentially with an increased risk of outcome due to effect on the liver => coagulation |
| Haemostatic drugs other than tranexamic acid |  |  |  | B02B, B02AB | Possible confounding factor: Same drug class as the exposure |
| Anti-coagulants |  |  |  | B01 | Possible confounding factor: Indicative during increased risk of outcome and may increase risk of bleeding and thereby exposure usage |
| Hereditary angioedema | The National Registry of Patients | 1994-2017 | ICD-10 & -8 | D841A | Indication for exposure; not part of the target population |
| Cancer |  | 1977-2017 |  | C, 14-20 | Possible confounding factor: Associated with increased risk of hypercoagulation and bleeding and thereby potentially associated with both outcome and exposure |
| Liver disease |  |  |  | K70-7, 570-3 |  |
| Thrombophilia |  | 1994-2017 |  | D685, D686 | Possible confounding factor: Associated with an increased risk of outcome and potentially with a decrease use of exposure due to the precaution of usage in women with thrombogenic risk factors |
| Haemophilia |  | 1977-2017 |  | D66, D67, D680, D681, D682, D683, D684, D688, D689, D69, 286, 287 | Possible confounding factor: May cause heavy menstrual bleeding and may influence coagulation |
| Blood transfusion |  | 1996-2017 | The Danish Classification System for Non-Surgical Procedures | BOQA | Possible confounding factor: Exposed are more likely to receive blood transfusion, and anaemia has been linked to increase risk of venous thrombosis |
| Hypertension* | The Prescrip-tion Registry | 1995-2017 | ATC code | C02A, C02B, C02C, C02DA, C02DB, C02DD, C02DG, C02L, C03A, C03B, C03D, C03E, C03X, C07A, C07B, C07C, C07D, C07F, C08, C08G, C09AA, C09BA, C09BB, C09CA, C09DA, C09DB, C09XA02, C09XA52 | Possible confounding factor: Associated with an increased risk of outcome and potentially with a decrease use of exposure due to the precaution of usage in women with thrombogenic risk factors |
| Diabetes Mellitus |  |  |  | A10 |  |
| Endometriosis | The National Registry of Patients | 1977-2017 | ICD-10 & -8 | N80, 6253 | Possible confounding factor: Associated with abnormal menstrual bleeding and thereby exposure usage and with increased risk of outcome (extreme cases of endometriosis may cause compression of veins; PCOS and hypothyroidism are associated with the metabolic syndrome; hyperthyroidism is associated with atrial fibrillation) |
| Polycystic ovary syndrome |  |  |  | E282, 25690 |  |
| Thyroid disease | Prescrip-tion Registry | 1995-2017 | ATC code | H03 |  |
| ***Confounding factors other than age and calendar time*** | | | | | |
| Pregnancy | The Medical Birth Registry &  The National Registry of Patients & The Registry of Legally Induced Abortion | 1973-2017  &  1977-2017  & 1973-2017 | ICD-10 | Each observation in the Danish Medical Birth Registry and The Registry of Legally Induced Abortion included as a pregnancy &  O00-8 | Not part of the target population and a  possible confounding factor: Associated with a decreased use of exposure and an increased risk of outcome |
| Surgery | The National Registry of Patients | 1977-2017 | NOMESCO | Each surgical procedure registered with a hospital stay of ≥24 hours  Uterine procedures KLC (except hysterectomies) | Possible confounding factor: Increases risk of outcome, and we could not detect exposure use during hospitalisation. |
| Hormonal contraception | Prescrip-tion Registry & The National Registry of Patients | 1995-2017 & 1996-2017 | ATC code & The Danish Classification System for Non-Surgical Procedures | G03AA, G03AB, G03AC, G03HB01 (except the product by the name Climen), G02BA03, G02BB01 &  (insertion of levonorgestrel-releasing intrauterine device in a hospital setting) BJCD01, BJCZ01 | Possible confounding factor: Alternative therapy options for heavy/abnormal menstrual bleeding and associated with increased risk of outcome |
| Systemic progestogens | Prescrip-tion Registry | 1995-2017 | ATC code | G03D |  |
| NSAIDs |  |  |  | M01A, except M01AX05 (glucosamine) |  |
| Educational level | The Edu-cation Registry | 1981-2017 | Elementary school only, secondary school only, skilled worker, theoretical education, theoretical education with research qualifications | | Possible confounding factor: Abnormal body weight, smoking, and low education are associated with increased risk of outcome and potentially with exposure usage through effect on risk of abnormal bleeding (weight and smoking) or medication use in general (education) |
| Body-mass index | The Medical Birth Registry | 2004-2017 | Weight in kg divided by height in m to power of two: <18.5, 18.5-25, >25-30, >30 | |  |
| Smoking status |  | 1991-2017 | Yes/no | |  |

** A woman was considered to have hypertension if she purchased two different types of antihypertensiva within 90 days, two calendar quartiles in a row (types of antihypertensiva: Adrenergic receptor antagonists, Diuretics, Vasodilators, Beta-blockers, Calcium channel blockers, Inhibitors of the renin-angiotensin system, Others*)

**Table S2:**

*Number of users of oral tranexamic acid and number of thromboses during usage in excluded and censored 15-49-year-old women according to the exclusion/censoring criterion*

| **Exclusion/Censoring criterion** | **No. of women*** | **No. of users of oral tranexamic acid** | **No. of thromboses during exposure to oral tranexamic acid**** | |
| --- | --- | --- | --- | --- |
|  |  |  | **Venous** | **Arterial** |
| Endometriosis | 24,046 | 3,996 | < 3 | 0 |
| Polycystic ovary syndrome | 14,805 | 1,645 | 0 | 0 |
| Thyroid disease | 64,671 | 7,020 | 0 | 0 |
| Diabetes mellitus | 42,344 | 5,575 | 0 | < 3 |
| Hypertension | 15,196 | 1,985 | 0 | 0 |
| Cancer | 39,904 | 4,141 | 0 | 0 |
| Liver disease | 8,697 | 981 | 0 | 0 |
| Haemophilia | 8,016 | 1,338 | 0 | 0 |
| Thrombophilia | 3,354 | 192 | 0 | 0 |
| Hereditary angioedema | 103 | 34 | 0 | 0 |
| Use of hormone therapy | 135,758 | 17,038 | 0 | < 3 |
| Use of anticoagulation medication | 40,314 | 5,093 | 0 | < 3 |
| Use of haemostatic drugs  other than tranexamic acid | 1,966 | 319 | 0 | 0 |
| Use of ulipristal acetate  for uterine fibroids | 800 | 331 | 0 | 0 |
| Blood transfusion | 14,502 | 1,983 | 0 | 0 |

** Only non-hysterectomised, non-oophorectomised women with no history of thrombotic disease were included in the counts.*

***Within five days plus one week following date of prescription redemption.*

*Counts lower than 3 are not allowed reported precisely due to the data protection rules.*

**Table S3:**

*Number of users of oral tranexamic acid and number of thromboses during usage during person-time that was temporarily censored in the study due to usage of hormonal contraception, systemic progestogen, or nonsteroidal antiinflammatory drugs/*

*Concomitant use of oral tranexamic acid and hormonal contraception or systemic progestogens or nonsteroidal antiinflammatory drugs.*

|  | **No. of users** | **No. of users of oral tranexamic acid** | **No. of thromboses during exposure to oral tranexamic acid*** | |
| --- | --- | --- | --- | --- |
|  |  |  | **Venous** | **Arterial** |
| Hormonal contraception |  |  |  |  |
| Combined oestrogen and progestin | 1,083,342 | 16,882 | 5 | 0 |
| Progestin-only | 385,022 | 13,157 | 0 | < 3 |
| Systemic progestogen | 132,284 | 10,519 | 3 | 0 |
| Nonsteroidal antiinflammatory drugs | 1,003,007 | 10,401 | < 3 | 0 |

**Within five days plus one week following date of prescription redemption.*

*Counts lower than 3 are not allowed reported precisely due to the data protection rules.*
